# Too much or never enough: a response to *Treatment of opioid disorders in Canada: looking at the ‘other epidemic’*

**DOI:** 10.1186/s13011-016-0076-z

**Published:** 2016-09-20

**Authors:** Joseph K. Eibl, Kristen A. Morin-Taus, David C. Marsh

**Affiliations:** 1 Northern Ontario School of Medicine, 935 Ramsey Lake Rd, Sudbury, Ontario P3E 2C6 Canada; 2 Canadian Addiction Treatment Centers, 13291 Yonge St., Ste. 403, Richmond Hill, Ontario L4E 4L6 Canada

## Abstract

Prescription opioid (PO) misuse is a major health concern across North America, and it is the primary cause of preventable death for the 18–35 year old demographic. Medication assisted therapy including methadone and buprenorphine, is the standard of care for patients with opioid-dependence. Moreover, both of these medications are recognized as essential medicines by World Health Organization. In Ontario Canada, the availability of medication assisted therapy has expanded substantially, with almost a ten-fold increase number of patients accessing methadone in Ontario in the past decade. In their manuscript, Fischer et. al. (2016), present a view that expansion of opioid maintenance therapy (OMT) has outpaced true patient need and alternate strategies should be considered as first-line treatments. Here, we present a countering perspective-that medication assisted therapy, along with other harm reduction strategies, should be widely available to all opioid-dependent people as first-line treatments.

## Background

Prescription opioid (PO) misuse is a major health concern across North America. Fueled by liberal PO prescribing during the late 1990’s and 2000’s, Ontario is often cited as a region suffering from a public health crisis due to PO-dependence [7, 11, 14]. Today, opioid-related overdose is the number one cause of death for 18–35 year olds in Ontario [12], and this trend holds true for Canada and the US [20]. Sadly, opioid-overdose deaths are far more likely to occur following abstinence-based treatment programs; and the reason is all too often relapse during a time of increased opioid sensitivity which follows detox [5]. In an effort to address the PO epidemic, some provinces in Canada have dramatically expanded access to medication assisted therapy, including the province of Ontario.

The evidence to support the efficacy of medication assisted treatment (e.g., methadone and buprenorphine) is well established for heroin-using patients [3], and due to the pharmacological similarities of prescription opioids (including oxycodone) methadone and buprenorphine have demonstrated efficacy for prescription opioid users as well [4]. However, Fisher et. al., (2016) also question the generalizability of studies conducted in a heroin-using population to the prescription opioid-dependent population.

Recently, the question has been raised, when is there too much medication assisted therapy? Fischer et. al., (2016) present a view that expansion of opioid maintenance therapy (OMT) has outpaced true patient need for treatment in the province of Ontario, Canada. Further, the authors imply that economic incentives surrounding delivery of methadone have ‘unduly influenced the expansion of OMT in Ontario’. Finally, the authors argue towards a continuum-of-care which focuses on alternatives to maintenance therapy citing taper, detox and abstinence-based approaches as alternatives for a subset of prescription-opioid dependent patients. Here, we argue against the position that OMT capacity has expanded beyond need; and we believe that significant need still exists, especially in Northern and rural regions of the province [9, 13].

## Commentary

For the majority of opioid-dependent patients, the evidence overwhelmingly supports OMT [4, 6, 16], and tapering strategies using methadone or buprenorphine have a very low probability of resulting in long term abstinence for opioid-dependent patients [18, 23]. In fact, we have previously shown that most patients who attempt to taper off methadone relapse within 12 months [19]. Fundamentally, the neurology of opioid-dependence is similar between heroin and other prescription opioids, and it is not comparable to other substance use disorders due to the profound neural re-wiring which occurs following prolonged opioid use [25]. The literature strongly supports OMT for opioid-dependence treatment of heroin and/or prescription opioid-dependence [16, 17]. Thus, OMT remains the clinical strategy with the best evidence for long term patient safety, social stabilization, and long-term health benefit – it is for this reason that both methadone and buprenorphine are recognized by the World Health Organization as essential medicines [24]. We are not arguing that methadone and/or buprenorphine are perfect, but for the majority of patients seeking treatment with the hope of stabilizing the impact dependence has had on their quality of life, OMT is the best option. In fact, a recent review of studies on OMT has shown this approach is even more effective for those using PO than for heroin users [15].

As suggested by Fisher et al., (2016), alternate approaches including tapering, counselling and psychosocial interventions may also have benefit; but again the evidence suggest that on their own, these treatment strategies are not as effective as OMT [1, 2]. With respect to programs which offer a combination of medication and counselling, evidence suggests that outcomes are not much better than OMT alone [1, 3, 21]. Other reports find some patients may benefit from coordination of OMT and psychosocial supports, but as Fisher et al., (2016) highlight, more research is needed to know which patients will benefit from more integrated treatment strategies [8]. Although a combination of psychosocial interventions and OMT may be as effective as OMT alone, Amato et al. [1] argue that provision of OMT should not be abandoned in the absence of resources for additional psycho-social treatment.

Quantifying the absolute need of OMT is challenging. However, reliable surrogates such as overdose-related deaths, overdose-related hospitalizations, and generalized opioid prescribing are all reasonable markers which support the burden and need for opioid-dependence treatment. Across North America there is an acknowledged need to expand methadone (and/or buprenorphine) programming [22]. Fisher et al., (2016) argue that Ontario’s methadone capacity exceeds that of the United States; yet, Norah Volkow, the Director of the National Institute for Drug Abuse in the US recently stated that expanding access to medication assisted treatment is a critical part of strategy to deal with the epidemic of prescription-opioid overdose in a recent article in NEJM [25]. Thus, comparing Ontario to the US does not seem a fair comparison.

In an earlier paper calling for expansion of MMT in Ontario, Fischer and colleagues used European countries and Australia, rather than the US, as a standard that would set the target for treatment availability in Ontario; a goal which is at least two-fold higher than the US comparison in the current paper [10]. Fisher et. al., (2016) also argue that the levels of OMT provision are disproportionate to other regions in Canada; however, British Columbia, Saskatchewan, and New Brunswick have a comparable proportion and patients receiving OMT (2015 public data).

Several factors limit our collective ability to expand methadone programming due to the resource intensive nature of observed dosing treatment. Perhaps the largest barrier to expansion is the availability of methadone trained addiction specialists. Recent advances in telemedicine have disrupted the most cumbersome barrier to expansion by allowing a given physician to service a number of specialized clinics in a broad geographic area both urban and rural via telemedicine; and Ontario is at the forefront of telemedicine-delivered OMT. We believe this a trend that will be adopted by other provinces and countries in the near future.

In the face of continued increases in opioid overdose deaths and other negative consequences of untreated opioid-dependence, OMT is still expanding in Ontario (and Canada); yet, there are still long wait lists and patients having to travel over 100 km to the nearest clinic in rural regions of the province [9]. Like most issues in medicine, we encourage policy makers, payers, and providers to use a comprehensive assessment of evidence across urban, rural, and remote geographies to inform measures which may disproportionately disadvantage patients who are already marginalized by geographic, political, and structural factors beyond their control.

## Conclusions

We support the Fisher et. al., (2016) recommendation that there is a need for more high quality epidemiological surveillance data to quantify and inform resource allocation towards treatment of opioid-dependence. However, we strongly oppose the implication that OMT programming is sufficiently serviced in the province of Ontario, especially in northern and rural regions. Moreover, the absence of robust data, which Fisher et. al., (2016) assert is lacking for other treatment options, is not a reason to reduce the availability of our most effective treatment at a time when people are dying from a public health crisis. It is our opinion that more resources should be allocated towards understanding and developing comprehensive care models which improve the overall health outcomes of this complex patient population - including expanded opioid maintenance treatment programming, especially in rural and remote regions of the country.
